# A Stable Cell Line Expressing Clustered AChR: A Novel Cell-Based Assay for Anti-AChR Antibody Detection in Myasthenia Gravis

**DOI:** 10.3389/fimmu.2021.666046

**Published:** 2021-07-08

**Authors:** Yu Cai, Lu Han, Desheng Zhu, Jing Peng, Jianping Li, Jie Ding, Jiaying Luo, Ronghua Hong, Kan Wang, Wenbin Wan, Chong Xie, Xiajun Zhou, Ying Zhang, Yong Hao, Yangtai Guan

**Affiliations:** Department of Neurology, Renji Hospital, School of Medicine, Shanghai Jiao Tong University, Shanghai, China

**Keywords:** myasthenia gravis, neuromuscular junction, clustered acetylcholine receptor, cell-based assay (CBA), stable cell line

## Abstract

Cell-based assays (CBAs) and radioimmunoprecipitation assay (RIPA) are the most sensitive methods for identifying anti-acetylcholine receptor (AChR) antibody in myasthenia gravis (MG). But CBAs are limited in clinical practice by transient transfection. We established a stable cell line (KL525) expressing clustered AChR by infecting HEK 293T cells with dual lentiviral vectors expressing the genes encoding the human AChR α1, β1, δ, ϵ and the clustering protein rapsyn. We verified the stable expression of human clustered AChR by immunofluorescence, immunoblotting, and real-time PCR. Fluorescence-activated cell sorting (FACS) was used to detect anti-AChR antibodies in 103 MG patients and 58 healthy individuals. The positive results of MG patients reported by the KL525 was 80.6% (83/103), 29.1% higher than the 51.4% (53/103) of RIPA. 58 healthy individuals tested by both the KL525 CBA and RIPA were all negative. In summary, the stable expression of clustered AChR in our cell line makes it highly sensitive and advantageous for broad clinical application in CBAs.

## Introduction

Circulating antibodies play a critical role in the pathogenesis and diagnosis of immune disorders. Myasthenia gravis (MG) is a prototype autoimmune receptor disease. The radioimmunoprecipitation assay (RIPA) is the most accurate and clinically available serological tests for autoantibodies, such as those specific for the acetylcholine receptor (AChR), providing laboratory confirmation for approximately 80% of patients with generalized MG (1). Muscle-specific receptor tyrosine kinase (MuSK) was shown to regulate the clustering of AChR, and anti-MuSK antibodies were first found in MG patients in 2000 (1, 2). Notably, despite similar electrophysiological manifestations and responses to cholinesterase inhibitors, approximately 10% of MG patients are double negative for both anti-AChR antibodies and anti-MuSK antibodies as measured by standard RIPA, a condition termed seronegative MG (SNMG) (3). Other autoantibodies, including those specific for low-density lipoprotein 4 (LRP4) and cortactin, are found in a certain percentage of SNMG cases but are less commonly found in MG than anti-AChR antibodies (4, 5).

In neuromuscular junctions, AChR is a pentamer consisting of α, β, γ and δ subunits (6) clustered in their native conformational state (7). Because it lacks the conformational epitopes of the native clustered AChR, solubilized AChR in RIPA fails to identify low-affinity clustered anti-AChR antibodies in SNMG patients (8). By coexpressing AChR subunits on the plasma membrane with rapsyn, a synaptic protein that induces AChR clustering, cell-based assays (CBAs) offer a highly natural membrane environment favoring binding between AChR and its circulating antibodies. Using a cell-based transient transfection assay, Leite et al. demonstrated the presence of serum clustered anti-AChR antibodies with low affinity in 66% of SNMG patients (8). Accumulating evidence has indicated improved sensitivity of CBAs for detecting clustered anti-AChR antibodies in MG patients deemed seronegative by non-cell-based assays (7, 9–16). Due to the costly and time-consuming nature of transient transfection, CBAs based on transient transfection are not commercially available for widespread clinical use for MG patients. Several groups have successfully generated cell lines stably expressing a single subunit of AChR to detect anti-AChR antibodies in patients (13, 17–20), but to our knowledge, no stable cell line expressing clustered AChR has been reported due to the limitation of expressing several large subunits and clustering protein in a single viral vector. There is thus an urgent need to provide an improved CBA that is time saving and cost effective. Here, we present a stable human embryonic kidney 293T (HEK 293T) cell line (KL525) expressing clustered AChR with rapsyn.

## Materials And Methods

### Patient Serum Samples

The participants in this study were 103 MG patients and 58 healthy individuals seen in Renji Hospital from August 2017 to June 2019. We estimated that with a sample size of 100 MG patients to receive both RIPA and CBA tests, the study would have more than 99% power to detect a between-group difference in detecting the level of circulating anti-AChR.

The whole blood was stored at 4°C for 12 hours to clot and then centrifuged at 1000 x gravitational units (g) for 10 minutes to separate the serum. The serum samples were frozen at -20°C.

MG was diagnosed by at least 1 of the following criteria (1): positive serum anti-AChR or anti-MuSK antibody confirmed by RIPA (9) (2); electromyographic characteristics of postsynaptic neuromuscular junction disorders; and (3) improvement of muscular weakness with cholinesterase inhibitors or immunosuppression.

The distribution and severity of muscular weakness was scored according to the Myasthenia Gravis Foundation of America (MGFA) classification system (21). Demographic data on all participants were collected. In addition, the results of anti-AChR and anti-MuSK antibody tests, thymectomy status and treatment history were also recorded for MG patients.

All the procedure performed in this study was approved by the Committee on Medical Ethics of Renji Hospital, Shanghai Jiao Tong University School of Medicine, and written informed consent was obtained from each subject.

### Lentivirus Construction of AChR Subunits

The human Rapsyn (*RAPSN*, BC004196), AChR subunit α1 (*CHRNA1*, BC006314) and β1 (*CHRNB1*, NM_000747) sequences were cloned into the CMV-MCS-PGK-Puro plasmid (Hanyin Biotech, Shanghai, China). The AChR subunit δ (*CHRND*, NM_000751) and ϵ (*CHRNE*, NM_00080) sequences were cloned into the CMV-MCS-PGK- Blasticidin plasmid (Hanyin Biotech, Shanghai, China). Endotoxin-free over-expression lentiviral vectors and original packaging vector plasmids were transfected into adherent-dependent epithelial-like cells. Enhancing buffer was added 12 hours later into the medium. After 4 hours, the medium was replaced with DMEM (Hyclone) containing 10% fetal bovine serum (FBS, Gbico) for cell growth. The supernatants were collected 2 days later and concentrated to obtain high titer lentivirus of AChR subunits with Rapsyn.

### Construction of AChR Overexpression Stable Cell Strain

Human embryonic kidney 293T (HEK 293T) cells were transfected with lentivirus expressing Rapsyn and AChR subunits α, β, δ, ϵ for 6 hours. The transfected HEK 293T were cultured in DMEM (Hyclone) with 10% FBS (Gbico) containing 5μg/ml puromycin and blasticidin at 37°C in the thermotank with humidified atmosphere of 5% CO_2_ for 2 weeks for the selection of our stable target strains. The medium containing puromycin (5 μg/ml) and blasticidin (5 μg/ml) was replaced every 2 days. The selected stable strains named as KL525 were verified by real-time PCR, western blotting and immunofluorescence.

### Real-Time PCR

Total RNA was extracted from cultured cells by Trizol reagent (Invitrogen) and reversed to cDNA using a RevertAid First Strand cDNA Synthesis Kit (Thermo Scientific Fermentas) following the manufacture’s instruction. Real-time PCR (RT-PCR) was performed using the SYBR Green RT-PCR Master Mix (Toyobo) on LightCycler 96 apparatus (Roche). The mRNA expression value was normalized to GAPDH and analyzed by ΔΔCT method.

Human CHRNA1-F: CTTAACTGGCCTGGTATTCTACC; Human CHRNA1-R: GCTCCACAATGACCAGAAGGAAC; Human CHRNB1-F: GTGTCGTGGTTCTCAACCTGC; Human CHRNB1-R: TAGACGCAGGTACAGCGGAAG; Human RAPSN-F: GGACAAAGGTGCTGGAGAAGAG; Human RAPSN-R: TGTCGATCTGGACCACAGCG; Human CHRND-F: TTGTCTACCACTACGGCTTCG; Human CHRND-R: GGTTCTCCTTGGCATCCTGT; Human CHRNE-F: GCCTGAGGATACTGTCACCATC; Human CHRNE-R: GTCCTTGCTGTAGTTGAGTCGG.

### Western Blot

Total proteins were extracted from the cultured KL525 cell strain using RIPA supplemented with protease inhibitor cocktail (Selleck) and electrophoresed on SDS-PAGE gel. The proteins were incubated with primary antibodies as follows: AChR α1 (1:500, ab221868, Abcam), AChR β1 (1:500, sc-166032, Santa cruz), AChR δ (1:500, sc-390896, Santa cruz), AChR ϵ (1:500, sc-376747, Santa cruz), Rapsn (1:1000, ab11423, Abcam) and β-actin (1:5000, A1978, Sigma-Aldrich).

### Immunofluorescence Staining

The KL525 cells were seeded on the glass coverslips (Fisherbrand) for 24 hours, fixed by 4% paraformaldehyde (PFA) for 20 minutes and washed 3 times with PBS. After blocked by 3% bovine serum albumin (BSA, sigma) with 0.2% Triton X-100 in 1×PBS for 1 hours, the cells were incubated with diluted serum (1:100) and AChR δ antibody (1:100, sc-390896, Santa cruz) at 4°C overnight. The secondary antibody conjugated with DyLight 488 (anti-human, 1:100, ab97003, Abcam) and Alexa Fluor 594 (anti-mouse, 1:100, 133880, Jackson ImmunoResearch) were incubated for 45 minutes at room temperature. Nuclei were counterstained with Prolong Gold antifade reagent with DAPI (P36935, Thermo Fisher Scientific).

Epifluorescence imaging was performed on Axio Vert.A1 microscope (Carl Zeiss) using a 20x objective. Confocal imaging was performed on LSM 710 microscope (Carl Zeiss) using a 40x objective (numerical aperture = 0.95) with a digital zoom of 5x and 1 air unit pinhole. The DyLight 488 fluorescence was excited with a 488 nm laser with a detection wavelength of 493-598 nm. The Alexa Fluor 594 fluorescence was excited with a 594 nm laser with a detection wavelength of 599-797 nm. The DAPI fluorescence was excited with a 405 nm laser with a detection wavelength of 410-507 nm. Images were acquired and processed with Zen software (Carl Zeiss). Each image was acquired on a single Z plane with minimal adjustment for brightness (whole-image adjustment).

### FACS Analysis Using KL525 Score

The KL525 cells were seeded at equal density (1 × 10^6^ cells/well) in 6-well plates. Cells were collected and centrifuged for cell-type analysis. After washing 3 times, the cells were resuspended in 100μl PBS and incubated with diluted serum (1:200) and AChR δ antibody (1:200, ab233758, abcam) for 20 minutes at room temperature. The corresponding 488-labeled anti-human secondary antibody (1:300, ab97003, Abcam) and APC-labeled anti-mouse secondary antibody (1:500, ab130786, Abcam) were used for indirect conjugation labeling staining. FACS was used to detect anti-AChR antibodies in human serum based on the KL525 score. The KL525 score was the percentage of gated (FITC-conjugated anti-human/APC-conjugated anti-mouse) double-positive cells in the second quadrant, excluding dead cells. The threshold KL525 score was 5.78, determined according to the mean + 2 SDs of the results from the negative controls. Cells were analyzed immediately using the BD FACS system (BD LSR II). Data were acquired and analyzed with the FlowJo 10.0 software.

### Statistical Analyses

Data are mean ± SD. Statistical differences were determined using either Student’s t test (2-tailed) or 2-way ANOVA followed by Tukey’s *post hoc* test, as appropriate. P values less than 0.05 were considered significant.

The power for this study is calculated based on a two-sided t-test with a significance level of 5%. With a sample size of 100 subjects with circulating anti-AChR antibodies detected by both RIPA and CBA based on KL525 cells, this study will have more than 90% power to detect a difference between RIPA and CBA in the proportion of subjects with positive circulating anti-AChR antibodies, given that the probabilities of positive circulating anti-AChR antibodies is 51.4% for RIPA and 80.6% for CBA.

## Results

### Establishment of Stable Cell Line Expressing Clustered AChR

**Figure 1:**
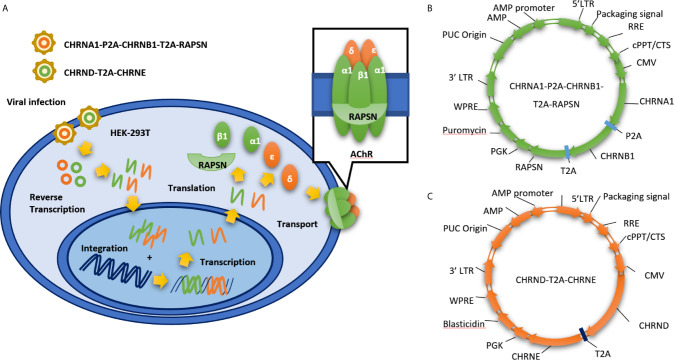
Establishment of stable cell line expressing clustered AChR *via* a dual lentiviral system. **(A)** Schematic of our stable cell line expressing clustered AChR (KL525). It is established by infecting HEK 293T cells with dual lentiviral vectors expressing the genes encoding the human AChR α1, β1, δ, ϵ and the clustering protein rapsyn. **(B)** CMV-MCS-PGK-Puro lentiviral vectors expressing human rapsyn and the AChR α1 and β1 subunits. **(C)** CMV-MCS-PGK-Blasticidin lentiviral vectors expressing the human AChR δ and ϵ subunits. LTR, long terminal repeat; RRE, Rev Response Element; cPPT/CTS, central polypurine tract and the central termination; CMV, Cytomegalovirus; CHRNA1, Cholinergic Receptor Nicotinic Alpha 1 Subunit; CHRNB1, Cholinergic Receptor Nicotinic Beta 1 Subunit; CHRND, Cholinergic Receptor Nicotinic Delta Subunit; CHRNE, Cholinergic Receptor Nicotinic Epsilon Subunit; RAPSN, gene encoding rapsyn; PGK, phosphoglycerate kinase; WPRE, Woodchuck Hepatitis Virus (WHP) Posttranscriptional Regulatory Element.

**Figure 2:**
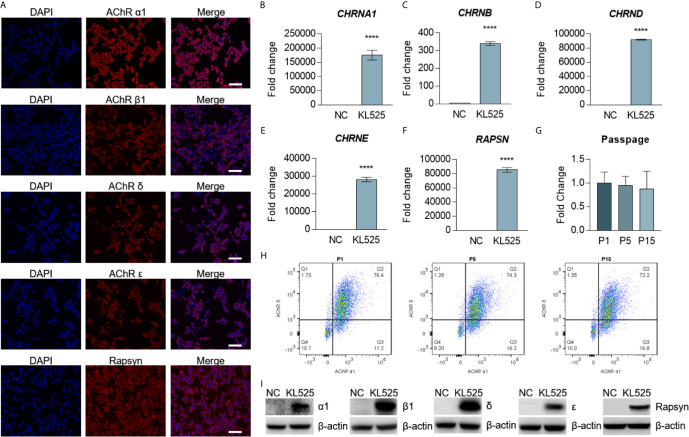
Stable expression of AChR subunits and rapsyn in HEK293T cells. **(A)** Double immunofluorescence staining with 4′,6-diamidino-2-phenylindole (DAPI, blue), AChR subunits (red) and rapsyn (red) in the stable cell line KL525. Bar, 100 μm. **(B–F)** mRNA expression of the human AChR subunit genes *CHRNA1*, *CHRNB*, *CHRND* and *CHRNE* and the AChR-clustering protein gene *RAPSN* in KL525 cells, as measured by real-time PCR. Uninfected HEK293T cells were used as the negative control (NC). **(G, H)** Flow cytometric analysis and representative results of coexpression of the AChR α1 & δ subunits in KL525 cells after passage. **(I)** Western blot analysis of AChR α1, β1, δ, ϵ subunits and rapsyn. Uninfected HEK293T cells were used as the NC. ^****^P < 0.0001 compared with NC.

To address the limitations of transient transfection approaches, we generated a stable cell line expressing clustered AChR by infecting HEK 293T cells **(** **Figure 1A** **)** with both CMV-MCS-PGK-Puro lentiviral vectors expressing human rapsyn and the AChR α1 and β1 subunits **(** **Figure 1B** **)** and CMV-MCS-PGK-Blasticidin lentiviral vectors expressing the human AChR δ and ϵ subunits **(** **Figure 1C** **)**. We selected the stable cell line by puromycin (5 μg/ml) and blasticidin (5 μg/ml). The expression of clustered AChR in KL525 cells was verified by immunofluorescence **(** **Figures 2A** and **S1** **)**, real-time PCR **(** **Figures 2B–F** **)** and Western blotting **(** **Figure 2I** **).** Flow cytometric analysis demonstrated a slight decreasing trend in coexpressed AChR α1 & δ subunits in KL525 cells at passages 1, 5 and 15, but no statistically significant difference was observed, indicating a stable expression of clustered AChR **(** **Figures 2G, H** **)**.

### Detection of Anti-AChR Antibodies in Patients With Myasthenia Gravis

**Figure 3:**
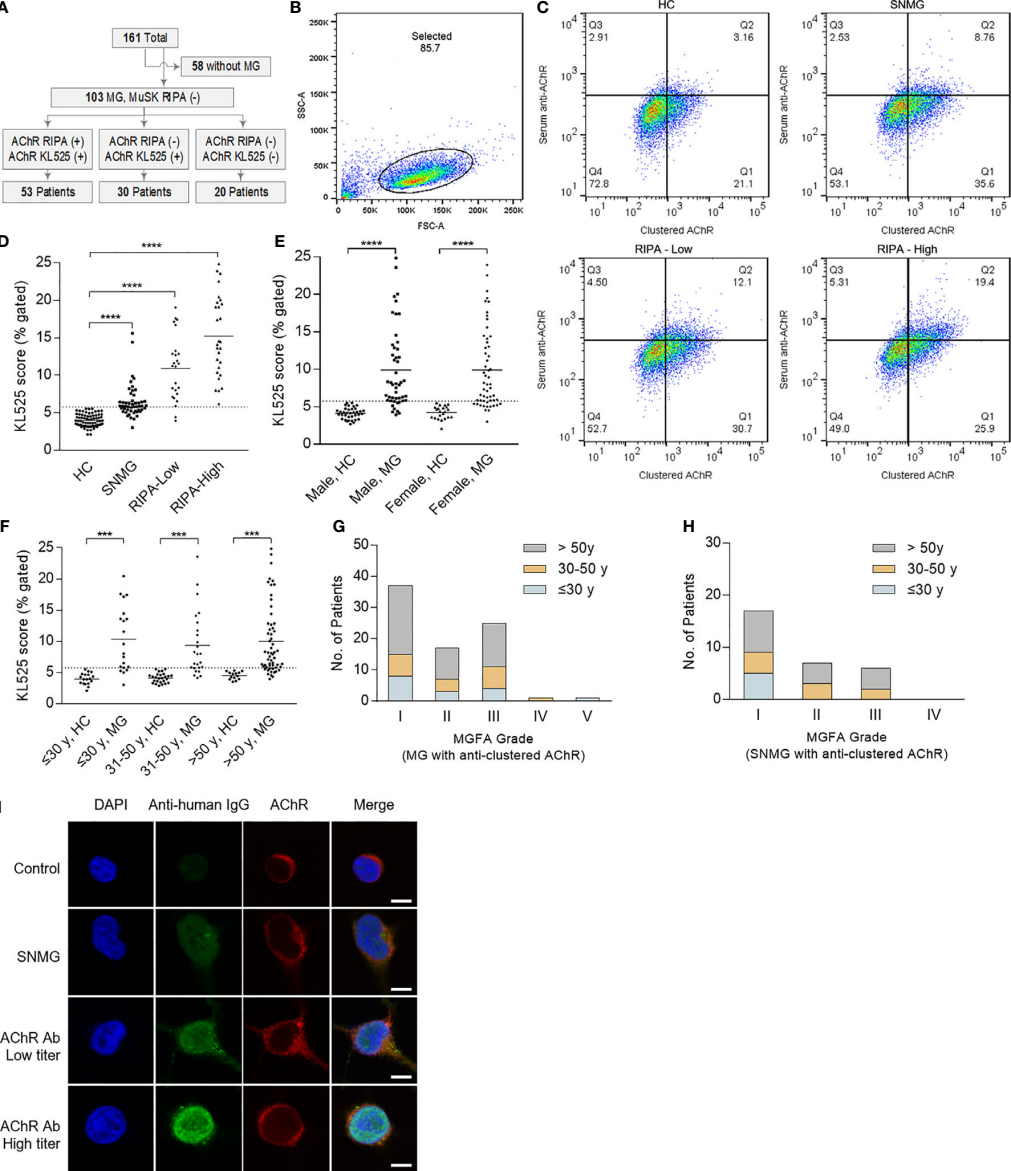
Detection of AChR in patients with myasthenia gravis. **(A)** Flow diagram showing participants in this study. The cell line stably expressing the clustered acetylcholine receptor (AChR) (KL525) identified anti-AChR antibodies in 30 of 50 (60.0%) radioimmunoprecipitation assay (RIPA)-seronegative myasthenia gravis (SNMG) patients. MG indicates myasthenia gravis, and MuSK indicates muscle-specific tyrosine kinase. **(B–D)** Representative results of FACS results and analysis of the KL525 score (double-positive cells for both AChR expression and serum antibody binding). The threshold KL525 score was 5.78, determined according to the mean + 2 SDs of the results from the negative controls. KL525 scores in healthy control group (HC, n = 58), in patients with positive for anti-AChR antibodies with a high RIPA titer (RIPA-High, RIPA≥8 nmol/L, n = 28), in patients with positive for anti-AChR antibodies with a low RIPA titer (RIPA-Low, 0.5 nmol/L<RIPA<8 nmol/L, n = 25) and in patients with SNMG (n = 50). **(E)** KL525 scores in male healthy control (HC) group, male MG patients, female healthy control group and female MG patients. **(F)** KL525 scores in healthy individuals less than or equal to 30 years old, MG patients less than or equal to 30 years old, healthy individuals in 30-50 age range, MG patients in 30-50 age range, healthy individuals older than 50 years old, MG patients older than 50 years old. **(G)** MGFA grade in MG patients with clustered anti-AChR antibody detected by KL525. **(H)** MGFA grade in SNMG patients with clustered anti-AChR antibody detected by KL525. **(I)** Representative immunofluorescence staining. Triple immunofluorescence staining with DAPI (blue), anti-human antibody (Anti-human, green) and anti-mouse antibody (Anti-mouse, red) in KL525 cells exposed to human serum. Anti-human secondary antibody was used to detect anti-AChR antibody in human serum, and anti-mouse antibody was used to detect mouse AChR δ antibody bound to clustered AChR expressed in KL525 cells. Bar, 10 μm. ^***^P < 0.001, ^****^P < 0.0001.

A total of 161 participants were recruited in this study, specifically, 103 (64.0%) with MG and 58 (36.0%) without MG **(** **Figure 3A** **)**. Among the 103 MG patients, 50 (48.5%) were RIPA-SNMG, and anti-AChR antibodies were detected by RIPA in 53 (51.5%). All healthy individuals were negative for anti-AChR antibodies by both the KL525 CBA and RIPA. A total of 83 of the 103 (80.6%) MG patients were positive for anti-AChR antibodies by the KL525 CBA **(** **Table 1** **)**. All patients who tested positive for anti-AChR antibodies were also confirmed by the KL525 CBA. Thirty-five (60.0%) RIPA-SNMG patients were positive for anti-AChR antibodies by the KL525 CBA **(** **Table 1** **)**. Patients with a higher RIPA titer of anti-AChR antibodies had higher KL525 scores **(** **Figures 3B–D** **)** and stronger immunofluorescence signals **(** **Figure 3I** **)** than healthy individuals. Despite the statistical significance of KL525 scores between healthy control (HC) group and MG patients, the groups of different sex and age did not show significant difference within MG or HC groups **(** **Figures 3E, F** **)**.

**Table 1:** Comparison of clinical features of RIPA-SNMG patients, RIPA-SNMG patients with anti-AChR antibodies detected by the KL525 CBA and patients with anti-AChR antibodies detected by both the KL525 CBA & RIPA.

Characteristics	RIPA-SNMG (n = 50)	RIPA-SNMG with anti-AChR antibodies detected by KL525 (n = 30)	Anti-AChR antibodies detected by both KL525 & RIPA (n = 53)
Sex, no. (%)			
Female	30 (60.0)	17 (56.7)	25 (47.2)
Male	20 (40.0)	13 (43.3)	28 (52.8)
Female/male ratio, no.	1.5/1	1.3/1	1.05/1
Age at onset, median (range), y	54 (9–86)	52 (9–86)	55 (4–85)
Clinical subtype, no. (%)			
OMG	26 (52.0)	17 (56.7)	22 (41.5)
GMG	24 (48.0)	13 (43.3)	31 (58.5)
Weakness distribution, no. (%)			
Ocular	43 (86.0)	25 (83.3)	46 (86.8)
Bulbar	3 (6.0)	1 (3.3)	10 (18.9)
Limb	21 (42.0)	11 (36.7)	22 (41.5)
Neck	3 (6.0)	2 (6.7)	7 (13.2)
Respiratory	0 (0.0)	0 (0.0)	3 (5.7)
Maximum MGFA grade, no. (%)			
I	26 (52.0)	17 (56.7)	22 (41.5)
IIa	12 (24.0)	7 (23.3)	9 (16.7)
IIb	0 (0.0)	0 (0.0)	1 (1.9)
IIIa	10 (20.0)	6 (20.0)	13 (24.5)
IIIb	2 (4.0)	0 (0.0)	6 (11.3)
IVa	0 (0.0)	0 (0.0)	0 (0.0)
IVb	0 (0.0)	0 (0.0)	1 (1.9)
V	0 (0.0)	0 (0.0)	1 (1.9)
Thymectomy, no. (%)	2 (4.0)	1 (3.3)	14 (26.4)
Immunosuppression, no. (%)	25 (50.0)	13 (43.3)	29 (54.7)

AChR, nicotinic acetylcholine receptor; GMG, generalized myasthenia gravis; MG, myasthenia gravis; MGFA, Myasthenia Gravis Foundation of America; OMG, ocular myasthenia gravis; SNMG, seronegative myasthenia gravis.

### Characteristics of Patients of RIPA-SNMG With Anti-AChR Antibodies Detected by KL525

Of the 30 patients with positive CBA and negative RIPA results, 17 (56.7%) were female. The median age at onset was 52 **(** **Table 2** **)**. Seventeen cases (56.7%) were ocular MG (OMG), and 13 (43.3%) were generalized MG (GMG). The predominant presentations were ocular (83.3%) and limb (36.7%) muscle weakness. Similar to MG patients with positive clustered anti-AChR antibody detected by KL525 **(** **Figure 3G** **)**, the major maximum MGFA grade in SNMG patients with positive clustered anti-AChR antibody was grade I (56.7%), followed by grades IIa (23.3%) and IIIa (20.0%) **(** **Figure 3H** **)**. Most patients responded well to either acetylcholinesterase inhibitors, corticosteroids, or both, but 5 (16.7%) required immunosuppression with azathioprine or tacrolimus, and 1 (3.3%) had a thymectomy. The pathological studies demonstrated thymoma in this 54-year-old female with MGFA grade IIIa disease. Compared with MG patients positive for anti-AChR antibodies by RIPA, RIPA-SNMG patients with anti-AChR antibodies detected by the KL525 CBA demonstrated reduced bulbar (3.3% *vs* 18.9%) and neck (6.7% *vs* 13.2%) muscle involvement, an absence of respiratory muscle weakness (0% *vs* 5.7%) and a reduced rate of thymectomy (3.3% *vs* 26.4%).

**Table 2:** Clinical characteristics of sNMG patients with positive anti-AChR antibodies detected by the KL525 CBA.

No.	Sex	Age at Onset	Muscular weakness	Clinical subtype	Maximum MGFA grade	Treatment	KL525 score
1	F	24	O	OMG	I	A	5.79
2	M	39	O	OMG	I	A+CS	5.79
3	M	61	O	OMG	I	A	5.81
4	F	43	O	OMG	I	CS	5.84
5	M	21	O	OMG	I	A	5.89
6	F	9	O	OMG	I	A	5.92
7	M	53	O	OMG	I	AZA	6.21
8	M	36	O	OMG	I	A+CS	6.31
9	F	29	O	OMG	I	A	6.52
10	F	73	O	OMG	I	A	7.35
11	M	59	O	OMG	I	No	8.09
12	F	64	O	OMG	I	A	8.21
13	M	86	O	OMG	I	No	8.48
14	F	23	O	OMG	I	No	9.58
15	F	32	O	OMG	I	No	9.87
16	F	61	O	OMG	I	A	14.41
17	F	80	O	OMG	I	A	15.59
18	M	76	L	GMG	IIa	No	5.86
19	F	34	O+L	GMG	IIa	A+CS	5.87
20	M	58	L	GMG	IIa	AZA+CS	6.31
21	M	63	O+L	GMG	IIa	A+AZA+CS	6.42
22	F	46	O+L	GMG	IIa	A+CS	6.62
23	M	57	L	GMG	IIa	A	7.56
24	M	45	O+L	GMG	IIa	A+CS	7.97
25	F	51	O+L+F	GMG	IIIa	A+CS+T	5.97
26	M	67	L	GMG	IIIa	A+CS	6.15
27	F	54	O+L	GMG	IIIa	AZA	6.25
28	F	60	N+L	GMG	IIIa	A	6.38
29	F	48	O+N+F	GMG	IIIa	A+CS	6.42
30	F	39	O+B	GMG	IIIa	No	9.16

A, acetylcholinesterase inhibitors; AChR, nicotinic acetylcholine receptor; AZA, azathioprine; B, bulbar muscle affected; CS, corticosteroids; F, facial muscle affected; GMG, generalized myasthenia gravis; L, limb muscle affected; M, methotrexate; MGFA, Myasthenia Gravis Foundation of America; N, neck muscle affected; O, ocular muscle affected; OMG, ocular myasthenia gravis; Thy, thymectomy; T, tacrolimus.

## Discussion

The detection of serum antibody is currently the most important serological diagnostic test for neuroimmune disorder such as MG. The antibodies commonly used in the diagnosis of MG are anti-AChR antibody and anti-MuSK antibody. Anti-AChR antibody accounts for approximately 85%, anti-MuSK antibody for only 8% and anti-LRP4 antibody for less than 5% of MG cases. Anti-Titin antibody is found in 6% of early onset of MG while anti-RyR is often absent in this stage. Anti-cortactin antibody can be detected in 23.7% of SNMG and 9.5% of seropositive MG (22–31). The remaining antibodies seen in MG, including those specific for LRP4, titin, RyR and cortactin, account for no more than 20% of MG cases. A few small-scale clinical studies have shown that these antibodies may be slightly more commonly seen in SNMG patients than in non-SNMG patients, but the diagnostic potential of these antibodies remains to be determined *via* large-scale clinical studies (5, 32–34).

Approximately 10% of all MG patients are reported to be SNMG patients who tested negative for both anti-AChR and anti-MuSK antibodies by the current clinically available RIPA and ELISA methods (1). However, accumulating evidence in the last decade has shown that approximately 50% of patients with SNMG have detectable circulating anti-AChR antibodies by CBA (8–10, 35, 36). Previous CBAs were established by transiently transfecting HEK293 cells with the subunits of adult or fetal AChR along with rapsyn-EGFP at a fixed ratio. Transiently transfected cells also express the subunits of AChR and rapsyn, which promotes the clustering of AChR and simulates the native conformation and structural epitope of AChR located at neuromuscular junctions. The transient transfection CBA is likely to generate between-batch deviations attributable to the effect of various factors, such as cell competence, transfection agents and plasmid quality, on the transfection efficiency (37). Additionally, the costly and time-consuming nature of transient transfection limits its clinical use.

Currently, no stable cell line expressing clustered AChR has been established due to the limitation of expressing several large subunits and clustering protein in a single viral vector. Our KL525 cell line stably expressing clustered AChR was established by lentiviral infection in HEK293T cells to overexpress AChR subunits with the AChR-clustering protein rapsyn. Flow cytometric data demonstrated no significant difference in the expression levels of clustered AChR in KL525 cells at passages 1, 5 and 15, indicating a stable expression of clustered AChR.

The positive results reported by the KL525 stable cell line for detecting anti-AChR antibodies in this study was 80.6%, 29.1% higher than the 51.4% of RIPA, the most sensitive clinical method. The positive results of anti-AChR antibody measurement by RIPA were reproducible in the KL525 stable cell line, and all healthy individuals tested by both the KL525 CBA and RIPA were all negative, suggesting that the KL525 stable cell line expressing clustered AChR could accurately reflect the known results of RIPA and sensitively identify low-affinity anti-AChR antibodies that were not detected by RIPA. Our results were consistent with those of previous CBA studies (8–10, 35, 36).

The major improvement in our CBA is that the stable expression of clustered AChR in this study was achieved by lentiviral infection while the transient expression of clustered AChR in previous CBAs was based on plasmid transfection. Our CBA with stable expression has several advantages over previous CBAs based on transient transfection: 1) Previous CBAs require fresh plasmid transfection into HEK cells each time before detection, while the expression of clustered AChR in our stable cell line is preserved with 15 passages without any further tedious, time and cost-consuming plasmid transfection procedure. 2) The stable expression in our KL525 cells minimizes possible between-batch deviations due to varied expression in repetitious transient transfection, resulting in better consistency of test results. 3) Furthermore, our stable cell line makes CBA embedded in microfluidic chip a feasible solution to provide a convenient, time- and cost-saving CBA for clinical use.

In summary, our CBA with cells stably expressing clustered AChR in this study demonstrated improved ability to detect low-affinity anti-AChR antibodies compared with RIPA. In comparison with its predecessor transient transfection CBA, our CBA has more advantages for wide clinical application due to its stable expression of clustered AChR, providing insight on establishing a CBA stably expressing complicated clustered cytoplasmic proteins with a dual lentiviral system.

## Data Availability Statement

The raw data supporting the conclusions of this article will be made available by the authors, without undue reservation.

## Ethics Statement

The studies involving human participants were reviewed and approved by the Committee on Medical Ethics of Renji Hospital, Shanghai Jiao Tong University School of Medicine. The patients/participants provided their written informed consent to participate in this study.

## Authors Contributions

YC, LH, DZ, and YG conceptualized and designed the study. YC, LH, JP, JLi, JD, JLu, RH, and KW acquired the data. YC, LH, and DZ analyzed and interpreted the data. YC and YG drafted the manuscript. JP, JLi, WW, CX, and XZ provided administrative, technical and material support. YZ, YH, and YG supervised this study. DZ and YG acquired the funding. All authors contributed to the article and approved the submitted version.

## Funding

This work was supported by the National Natural Science Foundation of China, Grant/Award Numbers [grant numbers 81771295 and 81230027]; and the Translational Medicine Collaborative Innovation Cooperation Research Project, Grant/Award [grant numbers TM201706 and TM201508].

## Conflict of Interest

The authors declare that the research was conducted in the absence of any commercial or financial relationships that could be construed as a potential conflict of interest.

## Supplementary Material

Supplementary Figure 1 Expression of AChR subunits and rapsyn in uninfected HEK293T cells. Double immunofluorescence staining with 4′,6-diamidino-2-phenylindole (DAPI, blue), AChR subunits (red) and rapsyn (red) in the stable cell line KL525. Bar, 100 μm.
