# Supplementary figures and images for: A Stable Cell Line Expressing Clustered AChR: A Novel Cell-Based Assay for Anti-AChR Antibody Detection in Myasthenia Gravis
Source: Front Immunol. 2021 Jul 8;12:666046. doi: 10.3389/fimmu.2021.666046 (PMC8297518; doi:10.3389/fimmu.2021.666046)

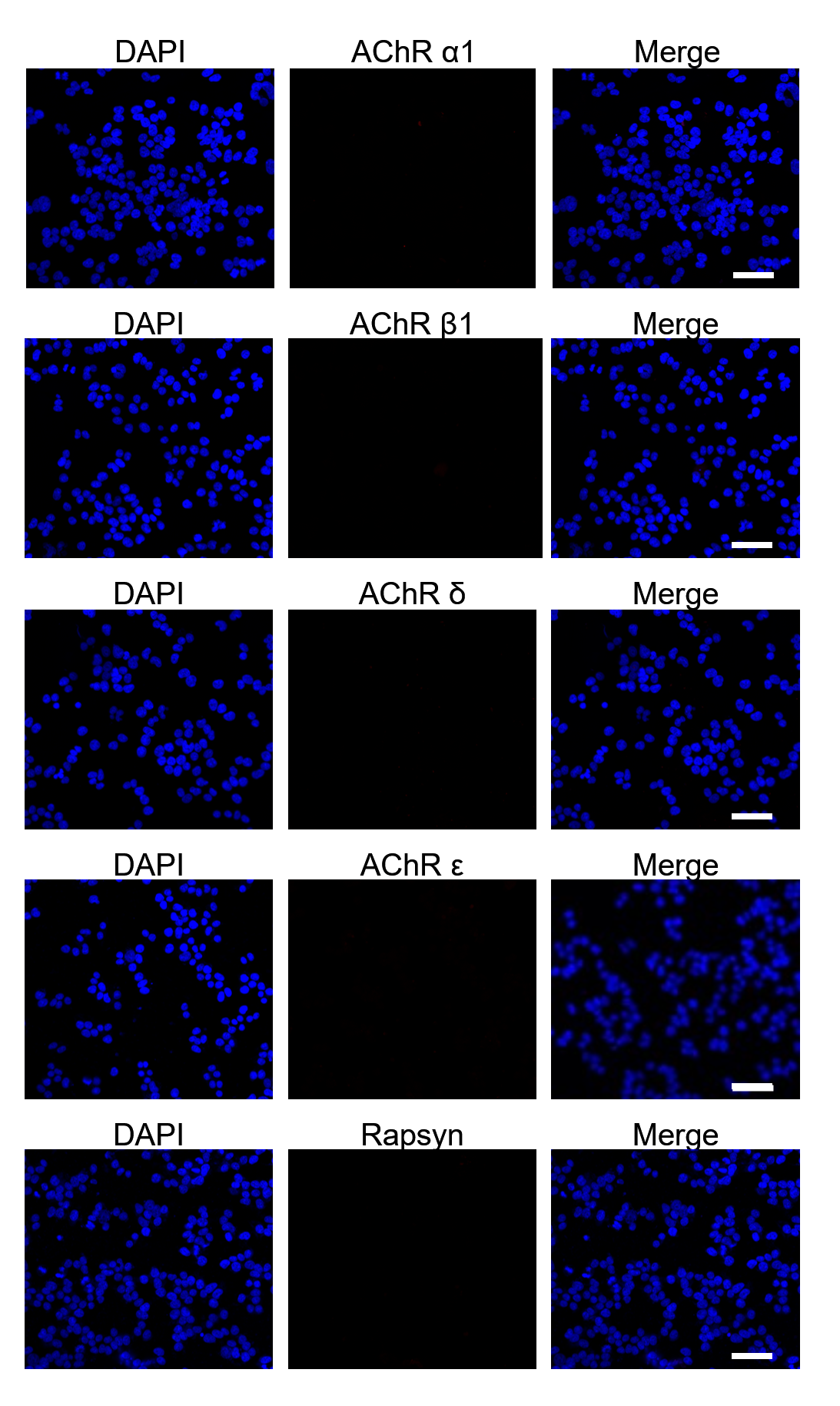

Supplement: Supplementary Figure 1 — Expression of AChR subunits and rapsyn in uninfected HEK293T cells. Double immunofluorescence staining with 4′,6-diamidino-2-phenylindole (DAPI, blue), AChR subunits (red) and rapsyn (red) in the stable cell line KL525. Bar, 100 μm. [file Image_1.tif]
